# The MAB-5/Hox family transcription factor is important for *Caenorhabditis elegans* innate immune response to *Staphylococcus epidermidis* infection

**DOI:** 10.1093/g3journal/jkae054

**Published:** 2024-03-13

**Authors:** Christopher Kywe, Erik A Lundquist, Brian D Ackley, Patrick Lansdon

**Affiliations:** Department of Molecular Biosciences, University of Kansas, Lawrence, KS 66045, USA; Department of Molecular Biosciences, University of Kansas, Lawrence, KS 66045, USA; Department of Molecular Biosciences, University of Kansas, Lawrence, KS 66045, USA; Department of Molecular Biosciences, University of Kansas, Lawrence, KS 66045, USA

**Keywords:** mab-5, immunity, gene expression, *Staphylococcus epidermidis*

## Abstract

Innate immunity functions as a rapid defense against broad classes of pathogenic agents. While the mechanisms of innate immunity in response to antigen exposure are well-studied, how pathogen exposure activates the innate immune responses and the role of genetic variation in immune activity is currently being investigated. Previously, we showed significant survival differences between the N2 and the CB4856 *Caenorhabditis elegans* isolates in response to *Staphylococcus epidermidis* infection. One of those differences was expression of the *mab-5* Hox family transcription factor, which was induced in N2, but not CB4856, after infection. In this study, we use survival assays and RNA-sequencing to better understand the role of *mab-5* in response to *S. epidermidis*. We found that *mab-5* loss-of-function (LOF) mutants were more susceptible to *S. epidermidis* infection than N2 or *mab-5* gain-of-function (GOF) mutants, but not as susceptible as CB4856 animals. We then conducted transcriptome analysis of infected worms and found considerable differences in gene expression profiles when comparing animals with *mab-5* LOF to either N2 or *mab-5* GOF. N2 and *mab-5* GOF animals showed a significant enrichment in expression of immune genes and C-type lectins, whereas *mab-5* LOF mutants did not. Overall, gene expression profiling in *mab-5* mutants provided insight into MAB-5 regulation of the transcriptomic response of *C. elegans* to pathogenic bacteria and helps us to understand mechanisms of innate immune activation and the role that transcriptional regulation plays in organismal health.

## Introduction

The innate immune system functions as an organism's first line of defense against pathogenic agents that overcome physical barriers of the host (e.g. cuticle/epidermis; Akira *et al*. 2006; Kumar *et al*. 2011). To protect against infections, an organism must not only recognize the invading microbe as foreign/pathogenic but also activate an appropriate systemic response to combat the pathogen. The “recognition-response” paradigm of the innate immune system is highly conserved across animal phyla (Ausubel 2005). In general, conserved microbial structures, termed pathogen-associated molecular patterns, are recognized by membrane-bound pathogen recognition receptors encoded by the host's genome (Kumar *et al*. 2011). Highly conserved components of signal transduction pathways [e.g. Toll-like receptors and nuclear factor kappa-ligh-chain-enhancer of activated B cells (NF-κB) transcription factors] translate the pathogen recognition signal into a cellular response via the activation of downstream effector molecules (Kopp and Ghosh 1995; Anderson 2000; Kawasaki and Kawai 2014). The specific effector molecules can vary between organisms but generally converge upon a set of evolutionarily conserved responses (e.g. reactive oxygen species generation, use of antimicrobial and antifungal peptides, and expression of bacterial-binding C-type lectins; McGreal *et al*. 2004; Muller *et al*. 2008; Kohchi *et al*. 2009; Koenderman *et al*. 2014).

The soil-dwelling nematode *Caenorhabditis elegans* is a tractable model system to examine host–pathogen interactions and investigate innate immune response to bacterial pathogens (Marsh and May 2012; Balla and Troemel 2013). *C. elegans* possess a robust immune response with evolutionarily conserved mechanisms for the detection and elimination of pathogens (Chavez *et al*. 2007; Schulenburg *et al*. 2008; Shivers *et al*. 2010; Marsh and May 2012; van der Hoeven *et al*. 2012; Dierking *et al*. 2016). However, core components of the innate immune response found in other organisms are either not involved in the innate immune response (Toll-like receptor) or are absent from the *C. elegans* genome entirely (NF-κB; Pujol *et al*. 2001; Couillault *et al*. 2004). The presence of a robust immune response suggests that *C. elegans* must utilize alternative pathways for immune activation, thus making nematodes an ideal model to study bacterial pathogenesis and identify additional genes that function in innate immunity.

Previous work has leveraged the genetic diversity of *C. elegans* wild isolates to investigate how genomic variation underlies pathogen avoidance and susceptibility (Schulenburg and Muller 2004; Reddy *et al*. 2009; Chang *et al*. 2011; Balla *et al*. 2015; Martin *et al*. 2017; Sterken *et al*. 2021). One such study found the Hawaiian wild isolate, CB4856, more susceptible to infection by the Gram-positive bacterium *Staphylococcus epidermidis* when compared with the lab-conditioned strain, N2 (var. Bristol; Lansdon *et al*. 2022). Additionally, gene expression analysis of CB4856 and N2 animals infected with *S. epidermidis* revealed that *mab-5* expression was significantly increased in N2, but not CB4856 animals.

The *mab-5* gene encodes for a Hox family transcription factor that is required for proper migration of Q neuroblasts (Chalfie *et al*. 1983; Kenyon 1986; Salser and Kenyon 1992; Maloof *et al*. 1999; Herman 2001; Chapman *et al*. 2008; Tamayo *et al*. 2013). In addition to their role in patterning the body plan, Hox genes are an important part of the *C. elegans* innate immune response. Notably, *egl-5* function is important for the nematode response to the Gram-positive bacterium *Staphylococcus aureus* and *Microbacterium nematophilum* (Gravato-Nobre *et al*. 2005; Irazoqui *et al*. 2008; Nicholas and Hodgkin 2009). Evidence of *mab-*5 upregulation in N2 animals infected with *S. epidermidis* raised the possibility that MAB-5 may be involved in activation of the innate immune response.

In this study, we show that the *mab-5* loss-of-function (LOF) mutations isolated in the N2 background are significantly more susceptible to *S. epidermidis* infection than wild-type animals or animals with a *mab-5* gain-of-function (GOF) mutation, *e1751*. We used high-throughput RNA-sequencing (RNA-seq) to identify changes in gene expression in N2 and *mab-5* GOF and *mab-5* LOF animals exposed to *S. epidermidis*. The differentially expressed genes (DEGs) identified provide insight into MAB-5 regulation of the transcriptomic response of *C. elegans* to pathogenic bacteria. We believe that the results of this project will provide a better understanding of the mechanisms of innate immune activation and the role that transcriptional regulation plays in organismal health.

## Methods

### *C. elegans* and bacterial strains

*C. elegans* and bacterial strains used in this study are listed in Supplementary Table 1. All strains were maintained at 20°C on nematode growth medium (NGM) plates seeded with *Escherichia coli* OP50 or the EVL2000 isolate of *S. epidermidis* (Brenner 1974; Do *et al*. 2022). All bacteria strains were grown at 37°C with shaking in low-salt Luria Bertani medium (10 g Bacto-tryptone, 5 g yeast extract, and 5 g NaCl).

### Survival assays

NGM plates were seeded with 20 µL of bacteria (*E. coli* or *S. epidermidis* EVL 2000) and incubated overnight at 37°C. Plates were acclimated to room temperature, and 30 late-stage L4 worms were transferred to the plate for each trial. Survival assays were performed at 20°C while transferring living worms to fresh plates and recording the number of dead worms daily. For each survival assay, a minimum of 3 replicates were collected for each worm strain and each pathogen.

### Exposure of nematodes to pathogens

NGM plates were seeded with 200 µL of bacteria. Approximately 1,000–2,000 L4 stage worms reared on *E. coli* OP50 were transferred to an NGM plate seeded with bacteria and incubated at 20°C for 24 h. After 24 h, worms were washed with M9 buffer 3 times to remove excess bacteria. Worms were suspended in 100 µL of M9 buffer and mechanically disrupted in liquid nitrogen using a ceramic mortar and pestle. Frozen tissue was transferred to a 1.5-mL microcentrifuge tube containing 1 mL of TRIZOL and flash frozen in liquid nitrogen and stored at −80°C until RNA isolation. For each worm and bacterial strain, 3 biological replicates were collected.

### RNA isolation and RNA-seq

Worm tissue was thawed on ice, vortexed for 5 s, and incubated at room temperature for 5 min. To each sample, 470 µL of chloroform was added, mixed by inversion, and phase separated for 2 min at room temperature. After centrifugation at 15,000 rpm at 4°C, the aqueous layer containing RNA (∼550 µL) was transferred to an RNase-free Eppendorf tube. Total RNA extraction was performed using the Monarch RNA Cleanup Kit (New England Biolabs) according to the manufacturer's instructions. Total RNA was quantified using the Qubit (ThermoFisher Scientific), and RNA quality was assessed using the TapeStation 4150 (Agilent Technologies). Sequence libraries were prepared using the NEBNext Ultra II Directional RNA Library Prep Kit for Illumina (New England Biolabs) and sequenced using paired-end 2 × 100-bp sequencing on the Illumina NextSeq 2000.

### RNA-Seq analysis and quantification of DEG

The quality of the 100-bp paired-end reads generated using the Illumina NextSeq 2000 was assessed using FastQC (Andrews 2010; v0.11.9). Illumina sequencing adapters and low-quality bases (Phred score <30) were trimmed from reads using Fastp (Chen *et al*. 2018; v0.20.0) with the “–correction” flag enabled. Reads were aligned to the *C. elegans* genome assembly (WormBase WS273 release) using HISAT2 (Kim *et al*. 2019; v2.1.0) with default settings. Aligned reads were mapped to the *C. elegans* annotated genome (WormBase WS273 release), and transcript abundances were quantified using featureCounts (Liao *et al*. 2014; v.2.0.0). Data normalization and DEG analysis were performed with DESeq2 (Love *et al*. 2014; v1.34.0) in R (v4.1.0) using a false discovery rate (FDR)-adjusted *P*-value < 0.05 and fold change ≥2 as cutoffs. Geneset enrichment analysis was performed with WormCat (Holdorf *et al*. 2020) using an FDR-adjusted *P*-value < 0.05 as the significance cutoff.

### Identification of MAB-5–binding sites near DEGs

Genomic coordinates of DEGs were obtained through WormBase (Howe *et al*. 2016). The coordinates were extended 1-kb upstream and downstream of the gene start and end sites to encompass potential MAB-5 binding sites outside of the coding region. Genomic coordinates of MAB-5 binding sites from mixed-stage (embryonic) and L2 animals were obtained from the ModERN database (Kudron *et al*. 2018). Analysis of genomic coordinates to detect overlap between DEGs and MAB-5 binding sites was performed with Microsoft Excel.

### Statistical analysis

All statistical analysis was carried out using SPSS Statistics 28.0.0.0 (IBM). The generalized Wilcoxon method was used for pairwise comparisons of survival curves and to calculate median survival (LT50). Chi-square test was used for assessing the statistical significance of the overlap in gene expression. Statistically significant differences are defined in the figure legends and noted in the figures with asterisks.

## Results

### *mab-5* LOF mutants exhibit increased susceptibility to *S*. *epidermidis* infection

**Fig. 1.**
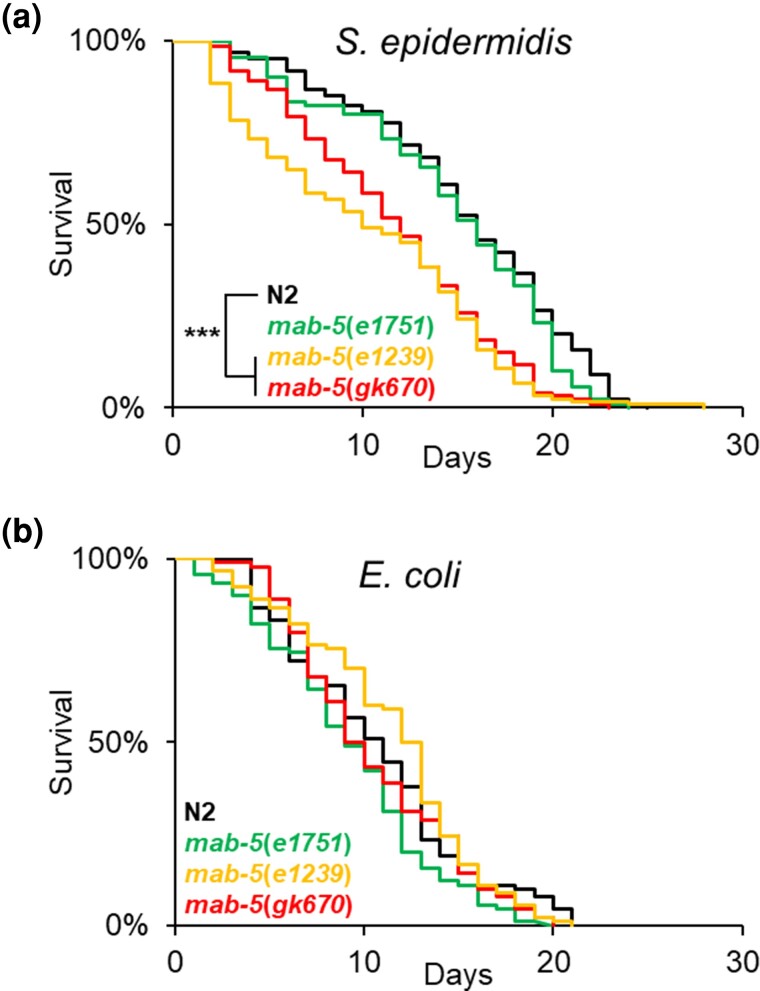
LOF *mab-5* mutants exhibit greater susceptibility to *S. epidermidis* exposure relative to N2. Survival of wild-type (N2) *mab-5* GOF mutant [*mab-5*(*e1751*)] and *mab-5* LOF mutants [*mab-5*(*e1239*); *mab-5*(*gk670*)] on NGM with a lawn of *S. epidermidis* a) or *E. coli* b). In each experiment, 30 worms were placed on the bacterial lawn and transferred daily while scored for survival. Curves represent a minimum of 3 independent experiments for each genotype. For each bacteria, statistical analysis was carried out by generalized Wilcoxon (mutant vs N2). ***P < 0.001.

To explore the role of *mab-5* in *C. elegans* innate immunity, we conducted survival analyses with the Gram-positive pathogen *S. epidermidis* using 2 *mab-5* LOF alleles, *e1239* and *gk670*, and the *mab-5* GOF allele, *e1751* (Kenyon 1986; Salser and Kenyon 1992; *C. elegans* Deletion Mutant Consortium 2012). Infection with the EVL2000 *S. epidermidis* strain found N2 and *mab-5* GOF animals to be similarly susceptible to infection with a median survival (LT_50_) of 15.3 ± 1.4 and 15 ± 0.6 days, respectively (Fig. 1a, Supplementary Table 2). In contrast, *mab-5* LOF animals were significantly shorter-lived after *S. epidermidis* infection relative to N2. *mab-5(e1239)* animals had a median lifespan of 10.5 ± 1.9 days (*P* < 0.001), whereas 50% of *mab-5*(*gk670)* animals were killed after an average of 11.5 ± 1.6 days (*P* < 0.001; Fig. 1a, Supplementary Table 2).

We also surveyed lifespan on nonpathogenic *E. coli* OP50, the standardized food for laboratory-reared *C. elegans*, to assess any general lifespan differences in *mab-5* mutants (Brenner 1974). On *E. coli*, the median lifespan of N2 animals was 10 ± 1.0 days. Perturbation of *mab-5* expression did not significantly affect lifespan on *E. coli* OP50, with 50% of the animals dying at 8.7 ± 0.7, 8.7 ± 1.5, and 11.0 ± 1.0 days for *mab-5*(*e1751*), *mab-5*(*gk670*), and *mab-5*(*e1239*), respectively (*P* > 0.05 vs N2; Fig. 1b, Supplementary Table 2).

We have previously reported findings whereby maintenance on the EVL2000 strain of *S. epidermidis* increases lifespan in N2 animals compared with those reared on *E. coli* OP50 (Lansdon *et al*. 2022), Not only did we corroborate this finding (N2 LT_50_: OP50 = 10 ± 1.0 days, EVL2000 = 15.3 ± 1.4; *P* < 0.001; Supplementary Table 2), but we observed *mab-5* GOF mutants to have a similar increase in lifespan on *S. epidermidis* compared with *E. coli*. *mab-5*(*e1751*) animals fed *S. epidermidis* had a median survival time of 15 ± 0.6 days, whereas 50% of animals reared on OP50 died after 8.7 ± 0.7 days (*P* < 0.001; Supplementary Table 2). Conversely, we found no statistically significant difference in *mab-5* LOF animals when comparing lifespan on *E. coli* with *S. epidermidis*. *mab-5*(*e1239*) animals had median lifespans of 11 ± 1.0 and 10.5 ± 1.9 days when reared on *E. coli* and *S. epidermidis*, respectively (*P* = 0.153), whereas the LT_50_ for *mab-5(gk670)* animals was 8.7 ± 1.5 and 11.5 ± 1.6 days when exposed to either *E. coli* or *S. epidermidis*, respectively (*P* = 0.103; Supplementary Table 2). Together, our results indicate that loss of *mab-5* function is detrimental to *C. elegans* survival after *S. epidermidis* exposure and that this decrease in survival is not due to a general decrease in lifespan. Since *mab-5(e1239)* exhibited the greatest difference in survival between N2 and *mab-5* LOF mutants, follow-up experiments used this allele.

### DEG among N2, *mab-5* LOF, and *mab-5* GOF exposed to *S. epidermidis*

Lifespan assays suggested that *mab-5* is important for *C. elegans* survival after *S. epidermidis* infection. To identify gene expression changes, N2 animals and *mab-5* mutants were exposed to either *S. epidermidis* or *E. coli* OP50 for 24 h followed by RNA-seq.

We first identified gene expression changes in response to *S. epidermidis* across strains (for all analyses, DEGs were defined as those with an FDR-adjusted *P*-value ≤ 0.05 and fold change ≥2, infected vs control). In total, we identified 401, 100, and 2778 DEGs by treatment (*S. epidermidis* vs *E. coli*) for N2, *mab-5*(*e1751*), and *mab-5*(*e1239*), respectively. In each genotype, we found that most genes were upregulated following *S. epidermidis* exposure compared with OP50 [N2, 370/401 = 92.3%; *mab-5(e1751)*, 85/100 = 85%; *mab-5(e1239*), 2408/2778 = 86.7%; Tables 1–3, Supplementary Tables 3–5].

**Table 1.** DEGs in N2 animals following 24-h exposure to *S. epidermidis*.

Condition	Total DEGs*^a^*	Total upregulated	Total downregulated	Total genes expressed*^b^*
*S. epidermidis*	401	370	31	15,980
*E. coli* OP50	—	—	—	15,394

^*a*^Genes with a FDR-adjusted *P*-value ≤0.05 and log_2_ fold change ≥1 relative to *E. coli* OP50.

^*b*^Total number of genes with >0 counts in each replicate.

**Table 2.** DEGs in mab-5(e1751) GOF animals following 24-h exposure to *S. epidermidis*.

Condition	Total DEGs*^a^*	Total upregulated	Total downregulated	Total genes expressed*^b^*
*S. epidermidis*	100	85	15	16,950
*E. coli* OP50	—	—	—	16,539

^*a*^Genes with a FDR-adjusted *P*-value ≤0.05 and log_2_ fold change ≥1 relative to *E. coli* OP50.

^*b*^Total number of genes with >0 counts in each replicate.

**Table 3.** DEGs in mab-5(e1239) LOF animals following 24-h exposure to *S. epidermidis*.

Condition	Total DEGs*^a^*	Total upregulated	Total downregulated	Total genes expressed*^b^*
*S. epidermidis*	2,778	2,408	370	17,144
*E. coli* OP50	—	—	—	16,797

^*a*^Genes with a FDR-adjusted *P*-value ≤0.05 and log_2_ fold change ≥1 relative to *E. coli* OP50.

^*b*^Total number of genes with >0 counts in each replicate.

**Fig. 2.**
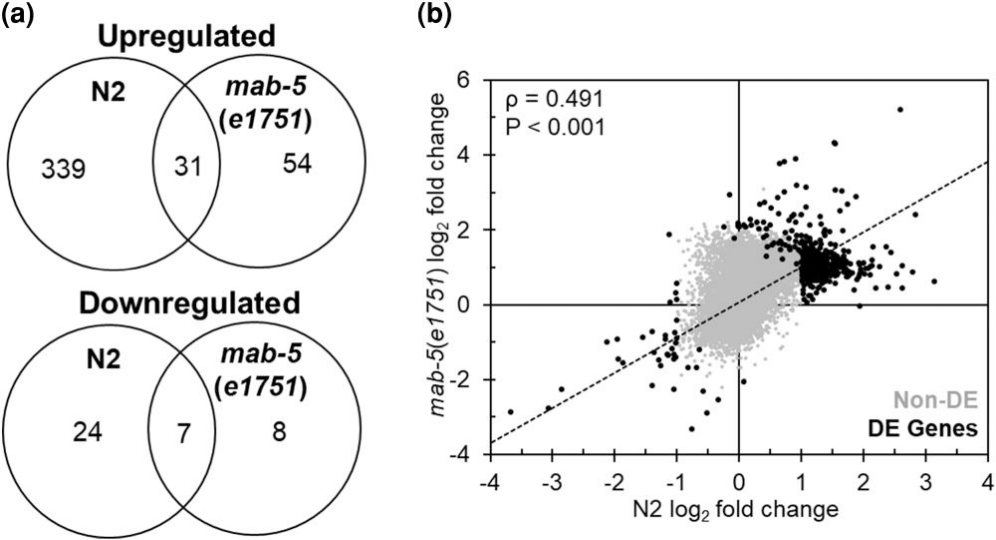
Expression differences in N2- and *mab-5*(*e1751*)-infected animals. a) Intersection of upregulated (top) and downregulated (bottom) genes in N2 and *mab-5*(*e1751*) animals infected with *S. epidermidis* EVL2000. b) Correlation between log_2_ fold changes in N2- and *mab-5*(*e1751*)-infected animals. Spearman's rho value and *P*-value are presented in the upper left corner. Gray (Non-DE) and black dots (DE Genes) represent expressed gene and DEG, respectively.

Next, we compared the lists of DEGs between N2 and *mab-5* GOF animals exposed to *S. epidermidis* to assess the degree of overlap in DEG between genotypes that had similar survival times following infection. Comparing upregulated genes between N2 and *mab-5* GOF animals fed *S. epidermidis*, a minority, 31 in total, overlapped between the 2 genotypes (N2-only, 339/370 = 92%; *mab-5* GOF-only 54/85 = 64%). Furthermore, the lack of overlap in differential expression was statistically significant (Χ^2^ = 46.35; *P* < 0.0001). In comparing downregulated genes between N2 and *mab-5* GOF animals fed *S. epidermidis*, again a minority, 7 in total, overlapped between the 2 genotypes (N2-only, 24/31 = 77%; *mab-5* GOF-only 8/15 = 53%). However, the lack of overlap in differential expression was not statistically significant (Χ = 2.77; *P* = 0.096; Fig. 2a, Supplementary Table 6). Notably, all DEGs shared between the 2 genotypes were expressed in the same direction. We also examined the correlation of gene expression using the log_2_ fold changes after infection. Although a minority of DEGs overlapped between N2 and *mab-5* GOF animals, expression levels were positively correlated between the 2 genotypes (*ρ* = 0.491; *P* < 0.001; Fig. 2b).

**Fig. 3.**
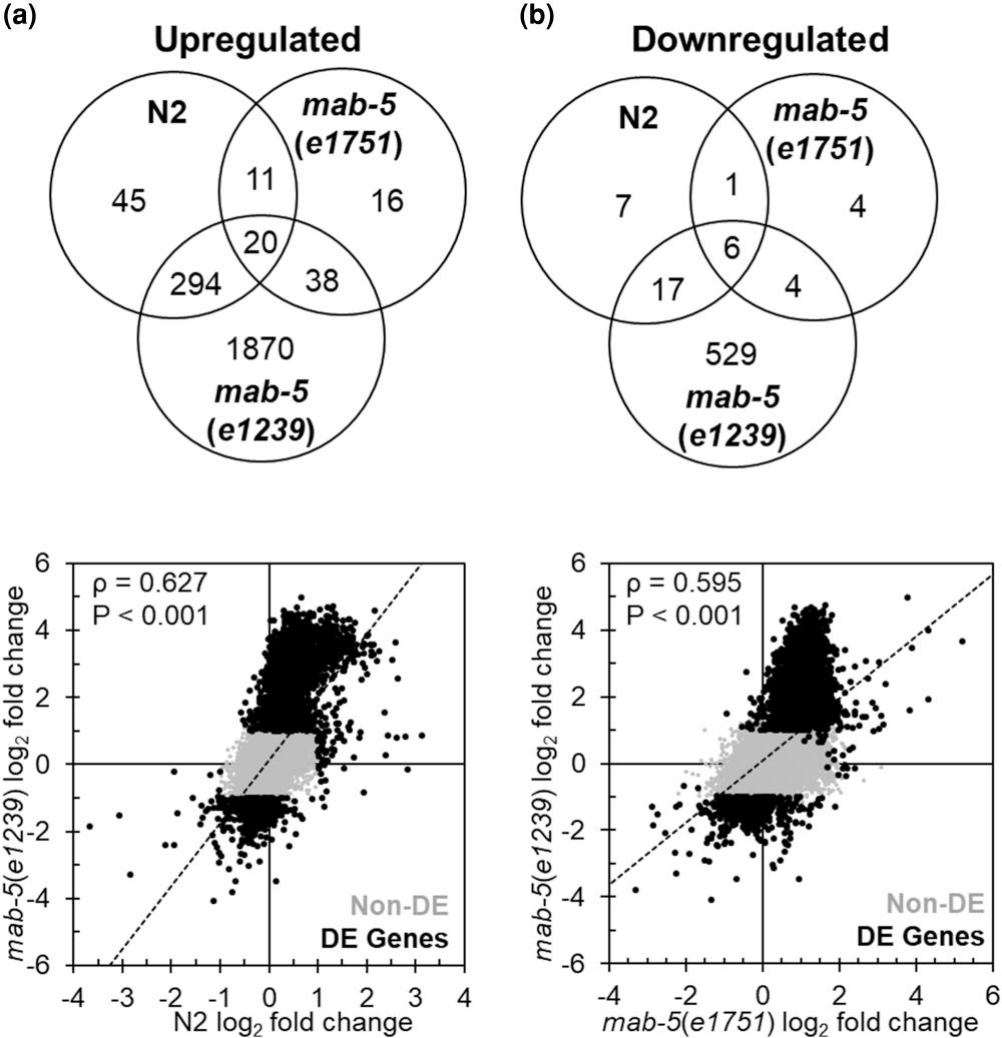
Expression differences in N2-, *mab-5*(*e1751*)-, and *mab-5*(*e1239*)-infected animals. a) Intersection of upregulated (left) and downregulated (right) genes in N2, *mab-5*(*e1751*), and *mab-5*(*e1239*) animals infected with *S. epidermidis* EVL2000. b) Correlation between log_2_ fold changes in N2- and *mab-5*(*e1239*)-infected animals (left) and *mab-5*(*e1751*)- and *mab-5*(*e1239*)-infected animals (right). Spearman's rho value and *P*-value are presented in the upper left corner. Gray (Non-DE) and black dots (DE Genes) represent expressed gene and DEG, respectively.

We performed tripartite Venn analysis on N2, *mab-5* GOF, and *mab-5* LOF animals after *S. epidermidis* exposure, again separating DEGs into upregulated and downregulated. In comparing upregulated genes, a majority of genes for both N2 and *mab-5* GOF animals overlapped with *mab-5* LOF (N2, 314/370 = 85%; *mab-5* GOF, 58/85 = 68%; N2 overlap, Χ = 847.78, *P* < 0.001; *mab-5* GOF overlap, Χ = 637.82, *P* < 0.001). In comparing downregulated genes among the 3 genotypes, we also observed a majority of genes shared between either N2 or the *mab-5* GOF mutants with the *mab-5* LOF mutant (N2, 23/31 = 74%; *mab-5* GOF, 10/15 = 67%; N2 overlap, Χ = 198.05, *P* < 0.001; *mab-5* GOF overlap, Χ = 176.17, *P* < 0.001; Fig. 3a, Supplementary Table 7). Within any overlap among genotypes, DEGs were expressed in the same direction, with the exception of one gene, *ech-9*, which was expressed in opposing directions (Supplementary Table 7). More specifically, *ech-9* was upregulated in N2, downregulated in *mab-5* LOF, and not differentially expressed in *mab-5* GOF animals. We also examined the correlation of gene expression for both N2 and *mab-5* GOF animals compared with *mab-5* LOF animals using the log_2_ fold changes after infection. In both instances, expression levels were strongly correlated between the 2 genotypes (N2 and *mab-5* LOF: *ρ* = 0.627, *P* < 0.001; *mab-5* GOF and *mab-5* LOF: *ρ* = 0.595, *P* < 0.001; Fig. 3b). Together, these results highlight the dramatic differences in gene expression between N2, *mab-5* GOF, and *mab-5* LOF animals exposed to *S. epidermidis*.

Although N2 and *mab-5* GOF worms were equivalently susceptible to *S. epidermidis*, their respective transcriptomic responses were relatively divergent, with a minority of genes in common. In contrast, *S. epidermidis* infection resulted in DEG profiles with considerable overlap between *mab-5* LOF animals and N2 and *mab-5* GOF animals, even though *mab-5* LOF animals were significantly more susceptible to *S. epidermidis* than animals with intact *mab-5* function.

### Geneset enrichment analysis among N2, *mab-5* GOF, and *mab-5* LOF exposed to *S. epidermidis*

Since *mab-5* LOF mutants exhibited survival deficiencies when compared with N2 animals or *mab-5* GOF mutants, we were interested in identifying biological processes that were enriched after *S. epidermidis* exposure. We first performed geneset enrichment analysis on DEGs that were exclusive to N2 (N2-only) and *mab-5(e1751)* (mab-5 GOF-only) and genes that were differentially expressed in both N2 and mab-5 GOF animals (N2 and *mab-5* GOF), separating genes based on whether they were upregulated or downregulated. Using upregulated genes, we found an enrichment in biological functions pertaining to the organismal stress response and immunity in both N2 and *mab-5* GOF animals (Table 4, Supplementary Table 8). The Gene Ontology (GO) terms “Major sperm protein,” “Extracellular material: collagen,” “Signaling: phosphatase,” “Cytoskeleton: microtubule,” and “Transmembrane protein” were enriched in N2-exlcusive DEGs, whereas genes corresponding to the GO term “Metabolism: lipid” were overrepresented in DEGs exclusive to *mab-5* GOF animals. Using downregulated genes, we found an enrichment of genes involved in lipid metabolism exclusive to N2 animals and genes encoding detoxification enzymes enriched in both N2 and *mab-5* GOF animals. No GO terms were enriched in the set downregulated genes exclusive to *mab-5* GOF animals. Together, these results indicate a common enrichment of biological processes related to stress response and innate immunity in N2 and *mab-5(e1751)* GOF animals.

**Table 4.** Enriched GO terms in N2 and mab-5(e1751) worms exposed to *S. epidermidis*.

Upregulated genes				
	GO term	Number of genes	percent*^a^*	FDR-adjusted *P*-value*^b^*
N2-only	Major sperm protein	21	67.7	5.3 × 10^−26^
	Extracellular material: collagen	25	13.6	1.3 × 10^−16^
	Signaling: phosphatase	17	8.7	1.7 × 10^−8^
	Cytoskeleton: microtubule	14	10.9	3.6 × 10^−8^
	Unassigned	132	2.1	5.7 × 10^−8^
	Transmembrane protein: unassigned	37	2.2	7.3 × 10^−3^
*mab-5*(*e1751*)-only	Metabolism: lipid	7	2.7	2.1 × 10^−3^
N2 and *mab-5*(*e1751*)	Stress response: pathogen	4	2.1	1.0 × 10^−3^
Downregulated genes				
N2-only	Metabolism: lipid	7	2.7	6.4 × 10^−6^
*mab-5*(*e1751*)-only	No enrichment of GO terms			
N2 and *mab-5*(*e1751*)	Stress response: detoxification	2	1.0	7.5 × 10^−3^

^*a*^Percentage of genes annotated with the indicated GO term, which are differentially expressed.

^*b*^FDR-adjusted *P*-value is a measure of enrichment (Fisher exact test; Bonferroni correction) of the GO term among DEGs.

We also performed geneset enrichment analyses on DEGs exclusive to N2, *mab-5*(*e1751*), or *mab-5*(*e1239*) animals, genes that were differentially expressed in all 3 genotypes, or DEGs identified in a combination of genotypes (N2 and *mab-5* GOF, N2 and *mab-5* LOF, and *mab-5* GOF and *mab-5* LOF) after *S. epidermidis* exposure, again separating genes based on upregulation or downregulation relative to an uninfected control (*E. coli* OP50). In upregulated genes exclusive to N2, the GO terms “Stress response: pathogen” and “Stress response: heavy metal” were enriched. In genes exclusive to *mab-5* LOF animals, the GO terms “Signaling: phosphatase,” “Transmembrane Protein,” “Extracellular material: collagen,” Signaling: Tyrosine kinase,” “Proteolysis, metallopeptidase,” and “Major sperm protein” were enriched. No GO terms were enriched for DEGS exclusive to *mab-5* GOF animals. The GO term “Stress response” was enriched in DEGs common to N2 and *mab-5* GOF animals, whereas the GO terms “Major sperm protein,” “Extracellular material: collagen,” “Signaling: phosphatase,” and “Cytoskeleton: microtubule” were enriched in N2 and *mab-*5 LOF animals. A single GO term, “Metabolism: lipid,” was enriched in *mab-5* GOF and *mab-5* LOF animals, whereas there was no enrichment of GO terms in genes upregulated in all 3 genotypes (Table 5, Supplementary Table 9).

**Table 5.** Enriched GO terms in upregulated N2, mab-5(e7151), and mab-5(e1239) worms exposed to *S. epidermidis*.

	GO term	Number of genes	percent*^a^*	FDR-adjusted *P*-value*^b^*
N2-only	Stress response: pathogen	4	2.1	3.9 × 10^−3^
	Stress response: heavy metal	2	12.5	5.3 × 10^−3^
*mab-5*(*e1751*)-only	No enrichment of GO terms			
*mab-5*(*e1239*)-only	Unassigned	747	11.8	8.3 × 10^−45^
	Signaling: phosphatase	81	41.3	7.6 × 10^−33^
	Transmembrane protein	259	15.3	5.7 × 10^−32^
	Extracellular material: collagen	63	34.2	5.4 × 10^−22^
	Cytoskeleton: microtubule	53	41.4	1.7 × 10^−21^
	Signaling: Y kinase	29	39.7	3.1 × 10^−11^
	Proteolysis: metallopeptidase	28	22.8	3.2 × 10^−6^
	Major sperm protein	10	32.3	8.1 × 10^−3^
N2 and *mab-5*(*e1751*)	Stress response	4	0.5	2.9 × 10^−3^
N2 and *mab-5*(*e1239*)	Major sperm protein	21	67.7	2.8 × 10^−27^
	Extracellular material: collagen	25	13.6	4.8 × 10^−18^
	Signaling: phosphatase	17	8.7	1.9 × 10^−9^
	Unassigned	120	1.9	3.1 × 10^−8^
	Cytoskeleton: microtubule	13	10.2	5.6 × 10^−8^
*mab-5*(*e1751*) and *mab-5*(*e1239*)	Metabolism: lipid	6	1.1	2.1 × 10^−3^
N2 and *mab-5*(*e1751*) and *mab-5*(*e1239*)	No enrichment of GO terms			

^*a*^Percentage of genes annotated with the indicated GO term, which are differentially expressed.

^*b*^FDR-adjusted *P*-value is a measure of enrichment (Fisher exact test; Bonferroni correction) of the GO term among DEGs.

In downregulated genes exclusive to N2, the GO term “Lipid: metabolism” was enriched. In genes exclusive to the *mab-5* LOF, the GO terms “Proteolysis proteasome: E3” and “Extracellular material: cuticlin” were enriched. There was no enrichment of genes exclusive to *mab-5* GOF animals. We also identified enrichment of genes related to lipid metabolism in N2 and *mab-5* LOF animals, whereas there was no enrichment of GO terms in genes shared by N2 and *mab-5* GOF animals or genes shared by *mab-5* GOF and *mab-5* LOF animals. An enrichment of C-type lectins was observed in genes downregulated in all 3 genotypes, N2, *mab-5*(*e1751*), and *mab-5*(*e1239*; Table 6, Supplementary Table 9). Thus, when considering genes that are upregulated in either N2 animals or *mab-5* GOF mutants but not *mab-5* LOF mutants following *S. epidermidis* infection, our results indicate an enrichment of biological processes related to stress response and innate immunity. When considering genes that are downregulated, our results indicate an enrichment of C-type lectins in all 3 genotypes.

**Table 6.** Enriched GO terms in downregulated genes identified in N2, mab-5(e1751), and mab-5(e1239) worms exposed to *S. epidermidis*.

	GO term	Number of genes	percent*^a^*	FDR-adjusted *P*-value*^b^*
N2-only	Metabolism: lipid	3	0.6	2.0 × 10^−3^
*mab-5*(*e1751*)-only	No enrichment of GO terms			
*mab-5*(*e1239*)-only	Proteolysis proteasome: E3	81	13.7	2.6 × 10^−39^
	Extracellular material: cuticlin	8	22.9	4.2 × 10^−5^
	Unassigned	167	2.6	8.5 × 10^−5^
N2 and *mab-5*(*e1751*)	No enrichment of GO terms			
N2 and *mab-5*(*e1239*)	Metabolism: lipid	4	0.8	2.9 × 10^−3^
*mab-5*(*e1751*) and *mab-5*(*e1239*)	No enrichment of GO terms			
N2 and *mab-5*(*e1751*) and *mab-5*(*e1239*)	Stress response: C-type lectin	2	0.8	9.0 × 10^−3^

^a^Percentage of genes annotated with the indicated GO term, which are differentially expressed

^*b*^FDR-adjusted *P*-value is a measure of enrichment (Fisher exact test; Bonferroni correction) of the GO term among DEGs.

### DEG in *mab-5* mutants relative to wild-type N2 animals on *E. coli* OP50

It is possible that perturbations in MAB-5 function are inducing expression of host response genes in the absence of a pathogen and, as a result, would not be detected in our earlier RNA-seq analysis. Using wild-type N2 animals as a baseline control, we identified gene expression changes in *mab-5* GOF and *mab-5* LOF mutants reared on nonpathogenic *E. coli* OP50 (for all analyses, DEGs were defined as those with an FDR-adjusted *P*-value ≤ 0.05 and fold change ≥2, *mab-5* mutant vs N2). In total, we identified 1472 and 167 DEGs for *mab-5*(*e1751*) and *mab-5*(*e1239*) mutants, respectively. In both GOF and LOF mutants, we found that most genes were upregulated compared with N2 [*mab-5(e1751)*, 1240/1472 = 84%; *mab-5(e1239*), 144/167 = 86%; Table 7, Supplementary Tables 10 and 11].

**Table 7.** DEGs in mab-5 mutants relative to wild-type N2 animals on *E. coli* OP50.

Genotype	Total DEGs*^a^*	Total upregulated	Total downregulated	Total genes expressed*^b^*
*mab-5*(*e1751*)	1,472	1,240	232	16,539
*mab-5*(*e1239*)	167	144	23	16,797
N2	—	—	—	15,394

^*a*^Genes with a FDR-adjusted *P*-value ≤0.05 and log_2_ fold change ≥1 relative to *E. coli* OP50.

^*b*^Total number of genes with >0 counts in each replicate.

We then performed geneset enrichment analyses on DEGs in *mab-5* GOF or *mab-5* LOF mutants, separating genes by updownregulation or downregulation relative to N2. In *mab-5*(*e1751*) DEGs, we did not observe enrichment of GO terms related to the host stress response or immune defense. Upregulated genes were enriched for the GO terms “Extracellular material: collagen,” “Signaling: phosphatase,” “Cytoskeleton: microtubule,” “Major sperm protein,” “Transmembrane protein,” “Signaling: Tyrosine Kinase,” and “Proteolysis general: metallopeptidase,” whereas downregulated genes were enriched for “Proteolysis proteasome: E3” and “Transcription factor: Zinc-finger” (Table 8, Supplementary Table 12). In *mab-5* LOF mutants, we found enrichment of the GO terms “Extracellular material: collagen” and “Stress response: pathogen” in upregulated genes, whereas there was no enrichment of GO terms in downregulated genes (Table 9, Supplementary Table 13). Together, our results suggest that loss of MAB-5 function activates a subset of host immune response genes in the absence of infection by *S. epidermidis*.

**Table 8.** Enriched GO terms in uninfected mab-5(e1751) worms.

	GO term	Number of genes	percent*^a^*	FDR-adjusted *P*-value*^b^*
Upregulated genes	Extracellular material: collagen	78	42.4	9.6 × 10^−44^
	Signaling: phosphatase	72	36.7	4.1 × 10^−37^
	Unassigned	479	7.6	4.9 × 10^−27^
	Cytoskeleton: microtubule	49	38.3	5.8 × 10^−26^
	Major sperm protein	28	90.3	3.6 × 10^−22^
	Transmembrane protein: unassigned	163	9.6	8.8 × 10^−19^
	Signaling: Y kinase	24	32.9	2.1 × 10^−11^
	Proteolysis general: metallopeptidase	16	13.0	8.1 × 10^−3^
Downregulated genes	Proteolysis proteasome: E3	41	7.0	1.6 × 10^−22^
	Transcription factor: ZF	6	11.3	2.5 × 10^−4^
	Unassigned	77	1.2	9.2 × 10^−3^

^*a*^Percentage of genes annotated with the indicated GO term, which are differentially expressed.

^*b*^FDR-adjusted *P*-value is a measure of enrichment (Fisher exact test; Bonferroni correction) of the GO term among DEGs.

**Table 9.** Enriched GO terms in uninfected mab-5(e1239) worms.

	GO term	Number of genes	percent*^a^*	FDR-adjusted *P*-value*^b^*
Upregulated	Extracellular material: collagen	12	6.5	1.2 × 10^−8^
	Stress response: pathogen	9	4.7	2.6 × 10^−5^
Downregulated	No enrichment of GO terms			

^*a*^Percentage of genes annotated with the indicated GO term, which are differentially expressed.

^*b*^FDR-adjusted *P*-value is a measure of enrichment (Fisher exact test; Bonferroni correction) of the GO term among DEGs.

### Expression differences and geneset enrichment analysis in N2-infected animals and uninfected *mab-5*(*e1239*) mutants

**Fig. 4.**
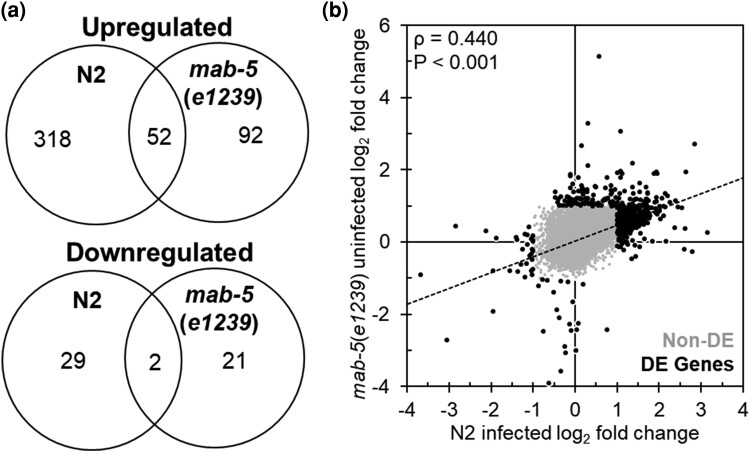
Expression differences in N2-infected animals compared with *mab-5*(*e1239*)-uninfected animals. a) Intersection of upregulated (top) and downregulated (bottom) genes in N2 animals infected with *S. epidermidis* and *mab-5*(*e1239*)-uninfected animals. b) Correlation between log_2_ fold changes in N2-infected and *mab-5*(*e1239*)-uninfected animals. Spearman's rho value and *P*-value are presented in the upper left corner. Gray (Non-DE) and black dots (DE Genes) represent expressed gene and DEG, respectively.

Because uninfected *mab-5* LOF mutants showed an enrichment in host defense genes, we were interested in examining the overlap in transcriptional responses between N2 animals exposed to *S. epidermidis* and uninfected *mab-5* LOF animals. Comparing upregulated genes between infected N2 and uninfected *mab-5* LOF animals, a minority, 52 in total, overlapped between the 2 (N2-only, 318/370 = 86%; *mab-5* GOF-only, 92/144 = 64%). Further the lack of overlap in gene expression was statistically significant (Χ = 31.25; *P* < 0.0001). In comparing downregulated genes, only 2 genes overlapped between the 2 genotypes (N2-only, 29/31 = 94%; *mab-5* GOF-only, 21/23 = 91%). However, the lack of overlap in differential expression was not statistically significant (Χ = 0.10; *P* = 0.756; Fig. 4a, Supplementary Table 14). Notably, all DEGs shared between infected N2 animals and uninfected *mab-5*(*e1239*) mutants were expressed in the same direction. We also examined the correlation of gene expression using the log_2_ fold changes for each condition. Although a minority of DEGs overlapped between N2 and *mab-5* LOF animals, expression levels were positively correlated between the 2 genotypes (*ρ* = 0.440; *P* < 0.001; Fig. 4b).

Separating genes based on upregulation or downregulation, we then performed geneset enrichment on DEGs exclusive to infected N2 animals (N2-only), exclusive to uninfected *mab-5* LOF mutants [*mab-5*(*e1239*)-only] or differentially expressed in both conditions [N2 and *mab-5*(*e1239*)].

In upregulated genes exclusive to N2-infected animals, we observed an enrichment of genes corresponding to the GO terms “Major sperm protein,” “Signaling: phosphatase,” “Extracellular material: collagen,” and “Cytoskeleton: microtubule”. In genes exclusive to uninfected *mab-5* LOF animals, we observed an enrichment in biological functions pertaining to the organismal stress response and immunity. In DEGs shared by infected N2 and uninfected *mab-5* LOF animals, we saw an enrichment of collagens and C-type lectins. Using downregulated genes, we found an enrichment of genes involved in lipid metabolism exclusive to N2 animals and genes encoding acid phosphatase enzymes enriched in both infected N2 and uninfected *mab-5* LOF animals. No GO terms were enriched in the set of downregulated genes exclusive to uninfected *mab-5* LOF animals (Table 10, Supplementary Table 15). Together, these data indicate that there are a handful of host immune genes whose expression is induced by loss of *mab-5* function in the absence of *S. epidermidis* infection, but the expression of these genes is not altered in wild-type N2 animals infected with *S. epidermidis*.

**Table 10.** Enriched GO terms when comparing gene expression in uninfected mab-5(e1239) animals with N2 worms exposed to *S. epidermidis*.

Upregulated genes				
	GO term	Number of genes	percent*^a^*	FDR-adjusted *P*-value*^b^*
N2-only	Major sperm protein	21	67.7	1.6 × 10^−26^
	Signaling: phosphatase	17	8.7	7.3 × 10^−9^
	Extracellular material: collagen	16	8.7	2.4 × 10^−8^
	Cytoskeleton: microtubule	13	10.2	1.7 × 10^−7^
	Unassigned	120	1.9	1.3 × 10^−6^
*mab-5*(*e1239*)-only	Stress response: pathogen	6	3.1	1.4 × 10^−3^
N2 and *mab-5*(*e1239*)	Extracellular material: collagen	9	4.9	1.8 × 10^−9^
	Stress response: C-type lectin	5	2.0	1.5 × 10^−3^
Downregulated genes				
N2-only	Metabolism: lipid	8	1.5	1.5 × 10^−6^
*mab-5*(*e1239*)-only	No enrichment of GO terms			
N2 and *mab-5*(*e1239*)	Lysosome: acid phosphatase	1	3.2	6.1 × 10^−3^

^*a*^Percentage of genes annotated with the indicated GO term, which are differentially expressed.

^*b*^FDR-adjusted *P*-value is a measure of enrichment (Fisher exact test; Bonferroni correction) of the GO term among DEGs.

### Overlap of known MAB-5–binding sites with pathogen-related DEGs

We next examined whether any of the DEGs corresponding to the enriched GO terms “Stress response: C-type lectin” or “Stress response: Pathogen” could be direct targets of MAB-5. In total, 23 genes with innate immune function were differentially expressed across our different gene expression analyses. For each gene, we obtained genomic coordinates comprising the coding region and 1-kb upstream and downstream of the start and end sites (Table 11). These coordinates were cross-referenced to known MAB-5–binding sites obtained from the ModERN database (Kudron *et al*. 2018). From this analysis, we found that none of the genomic coordinates of the 23 innate immune genes overlapped with MAB-5–binding sites cataloged in the ModERN database.

**Table 11.** Genomic coordinates of innate immune genes identified via geneset enrichment analysis.

Gene ID	Gene name	Chromosome	Gene start (bp)*^a^*	Gene end (bp)*^a^*
WBGene00020326	*math-38*	II	1576378	1580986
WBGene00018274	*F41C3.8*	II	4715046	4719536
WBGene00011979	*T24B8.5*	II	9081626	9084193
WBGene00014046	*clec-60*	II	10479043	10482874
WBGene00008492	*F01D5.1*	II	13995958	13998581
WBGene00006627	*tsp-1*	III	8237001	8240423
WBGene00009526	*clec-169*	IV	1260324	1265622
WBGene00016669	*ilys-2*	IV	2466678	2469879
WBGene00018971	*clec-67*	IV	3921165	3926039
WBGene00021581	*clec-70*	IV	3931708	3937307
WBGene00021582	*clec-71*	IV	3936458	3940979
WBGene00021583	*clec-72*	IV	3943816	3948362
WBGene00008584	*irg-4*	IV	12434833	12439236
WBGene00014132	*ZK896.1*	IV	12871776	12876360
WBGene00010125	*dod-22*	IV	12964374	12968392
WBGene00010123	*F55G11.2*	IV	12966445	12970289
WBGene00010124	*F55G11.4*	IV	12972624	12976603
WBGene00010745	*dod-17*	IV	12975303	12979343
WBGene00022261	*clec-210*	V	3117377	3121323
WBGene00009432	*cld-9*	V	13748181	13752891
WBGene00011844	*T19C9.8*	V	17238097	17241721
WBGene00017892	*F28B4.3*	X	3226501	3236408
WBGene00017582	*F18G5.6*	X	9261045	9265331

^a^Gene start and end sites comprise the coding region and 1-kb upstream and downstream of the start and end sites to encompass potential noncoding regulatory elements.

The lack of binding sites suggests an indirect relationship between *mab-5* function and expression of innate immune genes, perhaps through the differential expression of other transcription factors. We compared our DEG lists with a table of known *C. elegans* transcription factors (Supplementary File 1). In total, we identified 68 unique transcription factors that were differentially expressed, with 63 of 68 differentially expressed only in *mab-5*(*e1239*) LOF animals infected with *S. epidermidis* (Supplementary Table 16). Of the 68 transcription factors, 18 had binding site data in the ModERN database which we cross-referenced with the genomic coordinates of innate immune genes identified earlier (Table 11). Of 18, only one transcription factor, *pqm-1*, had any binding site overlap. Specifically, we observed *pqm-1*–binding sites overlapping the genomic coordinates of *F41C3.8*, *clec-72*, and *T24B8.5*. Together, these data suggest an indirect relationship between *mab-5* function and the expression of these innate immune genes, rather than any of the immune genes serving as direct targets of MAB-5 or as direct targets of a transcription factor differentially expressed in *mab-5* mutants.

## Discussion

### *mab-5* is required for prolonging the lifespan following *S. epidermidis* exposure

In a previous study, we found that *S. epidermidis* was pathogenic to CB4856 animals and shortened lifespan, whereas the lifespan of N2 animals was prolonged (Lansdon *et al*. 2022). These results suggested that the genomic variation between these isolates resulted in vastly different outcomes when they encountered *S. epidermidis.* Comparing gene expression differences between N2 animals and the Hawaiian isolate, CB4856, we noted that *mab-5* was upregulated in N2, but not CB4856. Upon reflection, *mab-5* was a good candidate to potentially underlie the differences in survival between N2 and CB4856 as Hox genes are known to contribute to innate immunity in *C. elegans* (Gravato-Nobre *et al*. 2005; Irazoqui *et al*. 2008; Nicholas and Hodgkin 2009).

We hypothesized that if *mab-5* was underlying the pathogenic outcomes observed in CB4586, then LOF *mab-5* mutants would be as susceptible to infection as CB4856 wild-type animals. In contrast, we found a more nuanced outcome. Specifically, in our survival assays, we found that animals lacking *mab-5* function had lost the lifespan extension effect of feeding *S. epidermidis* (compared with *E. coli*) observed in the wild-type N2 animals. In this context, it is important to note that all of the *mab-5* mutations used here were isolated in an otherwise N2 genetic background.

First, we used the *e1239* mutation, which was isolated in a forward genetic screen for male abnormal phenotypes. The *e1239* mutation is a strong LOF that molecularly affects a splicing site, leading to decreased MAB-5 protein levels. We confirmed our results using the *gk670* mutation, which was isolated in a different laboratory using a reverse genetic approach to create genetic deletion mutations in *C. elegans* genes. The *gk670* allele removes a significant portion of the *mab-5* coding region and introduces potential frameshifts that would prevent function of MAB-5 protein from being produced. The similarity in the susceptibility to *S. epidermidis* in the *e1239* and *gk670* animals indicates it is the LOF in *mab-5* rather than potential background differences. Further, we found that a GOF mutation, *e1751,* which was also isolated in a screen for male abnormal phenotypes, exhibited the same lifespan extension effects after feeding on *S. epidermidis* as wild-type N2 animals.

Taken together, we confirmed that in the N2 genetic background, feeding on *S. epidermidis* extends lifespan compared with feeding on *E. coli.* We concluded that intact *mab-5* function was required for the animals to derive the benefit of *S. epidermidis.* By itself, loss of *mab-*5 expression does not appear to be deleterious to these animals since lifespan on *E. coli* OP50 was not significantly different from N2 or *mab-5* GOF animals. Finally, we cannot specifically conclude that a failure to upregulate *mab-5* in the CB4856 background leads to the pathogenic outcome. Rather, there are likely to be additional genetic and/or genomic variation between these 2 isolates, which underlies the differences in survival.

### *S. epidermidis* induces stress-like pathways in the N2 background

There has been limited investigation into the host–pathogen relationship between *C. elegans* and *S. epidermidis*. Some reports suggest *S. epidermidis* can be pathogenic, while others, including our work, suggest it can be either pathogenic or beneficial. On the pathogen side, it is suggested that *S. epidermidis* accumulates in the intestine and relies in part on biofilm formation to kill the host (Begun *et al*. 2007). On the host side, research has exclusively used wild-type animals, focusing either on the expression of select immune genes, namely *atf-7* and *mpk-1*, or surveying for global transcriptional changes after *S. epidermidis* infection (JebaMercy *et al*. 2015; Lansdon *et al*. 2022).

We also used transcriptional analysis to better understand how *mab-5* might contribute to the interactions we observed between *S. epidermidis* and *C. elegans.* We compared gene expression profiles within genotypes between treatments, e.g. N2 on *E. coli* vs *S. epidermidis*, etc. as well as between genotypes, e.g. N2 vs *mab-5*(*e1239*) on *E. coli*, etc. Subsequently, we grouped DEGs and performed geneset enrichment analysis on sets of genes that were either exclusive to N2, *mab-5* GOF, or *mab-5* LOF animals, genes that were differentially expressed in all 3 genotypes, or DEGs identified in a combination of genotypes.

When we examined the genes that were differentially expressed across genotypes, we observed enrichment in at least 3 categories of genes that provide evidence that *C. elegans* activate stress-response pathways when shifted from *E. coli* to *S. epidermidis.* First, in our upregulated geneset, we saw a significant enrichment of genes encoding major sperm proteins. Major sperm proteins are the primary cytoskeletal components that enable sperm motility and promote oocyte maturation prior to fertilization (Roberts and Stewart 1995; Kuwabara 2003). Accelerated yolk production and an increased fertility rate have been associated with pathogenic infection in *C. elegans* (DePina *et al*. 2011). Additionally, when animals are starved or exposed to pathogenic stressors, sperm production as well as the prevalence of males increases to promote genetic diversity (Morran *et al*. 2009; Sharika *et al*. 2018).

Our analysis also detected an enrichment of genes encoding extracellular matrix (ECM) proteins, specifically collagens, in upregulated DEGs shared by N2 and *mab-5* LOF animals. Additionally, a separate set of collagen genes was enriched in upregulated DEGs exclusive to *mab-5* LOF animals. Collagens comprise both the cuticle and basement membrane of the ECM and are an integral component of the *C. elegans* epidermal barrier (Kramer 1994). Further, collagens have demonstrated roles in the host stress response as changes in collagen composition and collagen gene expression have been reported in animals experiencing innate immune and osmotic stressors (Ermolaeva and Schumacher 2014; Taffoni and Pujol 2015; Dodd *et al*. 2018; Mesbahi *et al*. 2020; Sandhu *et al*. 2021; Chandler and Choe 2022; Lansdon *et al*. 2022). Our findings provide additional support for collagens being associated with or possibly important for the *C. elegans* innate immune response, though it is not clear whether they are responsible for any observed differences in survival.

Additionally, an enrichment of genes involved in lipid metabolism was also identified. We found sets of lipid metabolism genes enriched in DEGs exclusive to either N2 or *mab-5* GOF animals. We also identified additional sets of lipid metabolism genes that were enriched in both the *mab-5* GOF and LOF mutants as well as a geneset enriched in both N2 animals and the *mab-5* LOF mutant. It is well-established that pathogenic infections can alter host metabolism and affect cellular pathways that control host nutritional status and lipid homeostasis (Anderson and Pukkila-Worley 2020). The enrichment of lipid metabolism in all 3 genotypes, albeit different sets of genes, suggests that the animals recognize *S. epidermidis* as a stressor and are modulating host metabolic processes in response. Looking specifically at N2 and *mab-5* GOF animals, we observed altered expression of genes involved in the synthesis of polyunsaturated fatty acids (*elo-6*), lipid transport (*lbp-8*, *vit-1*, *vit-3*, *vit-4*), triglyceride mobilization (*lipl-5*), and fatty acid degradation (*lipl-1*, *lipl-2*, *acdh-2*, *W03F9.4*). We also observed differential expression of genes involved in sphingolipid metabolism, with an upregulation of genes encoding anabolic processes (*gba-4*) and a downregulation of genes encoding catabolic processes (*asah-1*). Lipid metabolism also has a demonstrated role in the induction of the innate immune response. Nematodes with LOF mutations in elongase (*elo*) and desaturase (*fat*) genes exhibit greater susceptibility to pathogenic microbes (Nandakumar and Tan 2008; Anderson *et al*. 2019). Lipase-like (*lipl*) genes are expressed in response to nutritional stress conditions, and in the case of *lipl*-5, expression is altered upon exposure to pathogenic bacteria (Mallo *et al*. 2002). Further, the suppression of sphingolipid catabolism and the synthesis of sphingolipid-derived signaling molecules have been shown to increase *C. elegans* immunity toward pathogenic microbes (Lee *et al*. 2020; Nasrallah *et al*. 2023).

Last, of the DEGs that overlapped in N2, *mab-5* GOF, and *mab-5* LOF animals, all but one was expressed in the same direction. The *ech-9* gene encodes an enoyl-CoA hydratase that is essential for metabolizing fatty acids. It was found to be upregulated in N2 animals, downregulated in *mab-*5(*e1239*), and not differentially expressed in *mab-5*(*e1751*). Differential expression of *ech-9* has been previously reported following exposure to the Gram-negative pathogen, *Pseudomonas aeruginosa*, as well as the Gram-positive pathogens, *St. aureus* and *M. nematophilum* (Irazoqui *et al*. 2010; Pellegrino *et al*. 2014). Our transcriptomic data indicate that expression of *ech-9* may also impact lipid metabolism and the ability of *C. elegans* to survive following *S. epidermidis* infection.

Overall, the analysis of genes induced across animals fed *S. epidermidis* compared with *E. coli* suggests that the animals recognize *S. epidermidis* as a stressor and that the phenotypic outcomes are likely after the recognition event. Further, the differential expression of specific lipid metabolism genes exclusively in N2 and *mab-5* GOF animals suggests a potential role for their involvement in mounting an immune response to *S. epidermidis* infection.

### Increased survival on *S. epidermidis* is associated with functional categories corresponding to pathogen detection and response

Our analysis identified an enrichment of genes encoding C-type lectins and proteins involved in pathogen response. C-type lectins are a superfamily of proteins that contribute to the recognition of pathogenic agents in both vertebrates and invertebrates with well over 200 family members in *C. elegans* (Zelensky and Gready 2005; Schulenburg *et al*. 2008). Lectins exhibit differential upregulation after pathogen exposure with only a few upregulated across multiple pathogen species, suggesting a response that is specific to the pathogen rather than the pathogen type (e.g. Gram-negative vs Gram-positive; Troemel *et al*. 2006; Alper *et al*. 2007; Wong *et al*. 2007; Schulenburg *et al*. 2008). We observed a downregulation of 2 lectins, *clec-169* and *clec-210*. in all 3 genotypes after *S. epidermidis* exposure. The literature regarding *clec-169* and *clec-210* is limited but shows both lectins are induced in response to infection by the Gram-negative pathogen, *Klebsiella pneumoniae* (Yang *et al*. 2023). Their downregulation suggests a lack of involvement in the response to *S. epidermidis* exposure. Two additional lectins, *clec-60* and *clec-71*, were upregulated exclusively in N2 and *mab-5* GOF animals. Upregulation of *clec-60* has been observed in response to infection with Gram-positive pathogens such as *Enterococcus faecalis* and *St. aureus*, whereas infection with Gram-negative pathogens such as *P. aeruginosa* leads to repression of *clec-60* (O’Rourke *et al*. 2006; Wong *et al*. 2007; Irazoqui *et al*. 2010; Head and Aballay 2014). Changes in *clec-71* expression have been observed following exposure to *Cutibacterium acnes,* a Gram-positive bacterium found on the surface of the skin and other epithelial linings (Huang *et al*. 2020; Mongaret *et al*. 2021).

The upregulation of *clec-60* and *clec-71* expression in both N2 and *mab-5* GOF animals coupled with previous literature showing an induction of expression after infection with other Gram-positive pathogens raises the possibility that *clec-60* and *clec-71* are downstream targets of *mab-5* and may enable *C. elegans* to recognize and respond to *S. epidermidis*.

Geneset enrichment analysis also identified 2 pathogen response genes, *dod-22* and *F55G11.2*, that were upregulated in both N2 and *mab-5* GOF animals but not differentially expressed in *mab-5*(*e1239*) mutants. Both *dod-22* and *F55G11.2* encode for proteins containing complement C1r/C1s, Uegf, Bmp1 (CUB)-like domains. CUB domain proteins possess a diverse array of functions, ranging from complement activation and the innate immune response to developmental patterning and cell signaling (Bork and Beckmann 1993; Perry *et al*. 2007; Bolz *et al*. 2010; Simonsen *et al*. 2012; Ermolaeva and Schumacher 2014; Fanelli *et al*. 2023). Additionally, *dod-22* and *F55G11.2* have previously been shown to be activated following exposure to Gram-negative pathogens, such as *P. aeruginosa*, *Burkholderia pseudomallei*, and *Vibrio cholerae* (Sahu *et al*. 2012; Lee *et al*. 2013; Liu *et al*. 2016). Our findings demonstrate that these immune genes are also upregulated in response to *S. epidermidis*, suggesting that they may contribute to defense against Gram-positive pathogens as well.

### Loss of *mab-5* induces expression of stress response genes in the absence of infection

We identified DEGs in both uninfected *mab-5*(*e1239*) and *mab-5*(*e1751*) animals on *E. coli* OP50 (vs N2 var. Bristol) and performed geneset enrichment analysis. In uninfected *mab-5* GOF animals, we found enrichment of several categories identified in previous analyses, including enrichment of genes encoding collagens and major sperm proteins, but did not observe enrichment of GO categories corresponding to the organismal stress response. In contrast, uninfected *mab-5* LOF animals exhibited an enrichment of genes, 15 in total, that are typically induced following exposure to pathogenic bacteria. In comparing expression of these 15 genes with DEGs from uninfected *mab-5* GOF animals, we identified only 3 genes, *clec-60*, *dod-17*, and *F55G11.4*, that were also differentially expressed. Thus, in the absence of infection, loss of *mab-5* function leads to upregulation of a subset of immune response genes. However, a similar change in expression of immune genes was not observed in *mab-5* GOF animals, indicating that by itself, constitutive activation of *mab-5* is not inducing a pathogen-related stress response.

By identifying DEGs using different baselines of gene expression (*S. epidermidis* vs *E. coli* OP50; *mab-5* LOF vs N2, etc.), we identified several independent sets of immune genes enriched in different conditions, comprising a total of 23 genes. Of these genes, only 4, *F18G5.6*, *F41C3.8*, *F01D5.1*, and *F28B4.3*, were upregulated in either N2 or *mab-5*(*e1751*) animals infected with *S. epidermidis* but were not differentially expressed in *mab-5*(*e1239*) animals, regardless of infection status. These 4 genes remain functionally uncharacterized, and we were unable to find evidence in the literature of their involvement in the *C. elegans* immune response. Two genes, *F01D5.1* and *F18G5.6*, encode short proteins (<150 amino acids), suggesting a potential role as antimicrobial peptides. To shed some light on their function, we used SignalP 6.0 to predict the presence of signal peptides as *C. elegans* antimicrobial peptides contain a signal peptide on the N-terminus of the protein (Dierking *et al*. 2016; Teufel *et al*. 2022). The protein sequence of *F01D5.1* contained a cleavable signal peptide, whereas the sequence of *F18G5.6* did not, suggesting that *F01D5.1* may function as an antimicrobial peptide. The *F28B4.3* gene encodes a much longer protein (>2000 amino acids) and is predicted to contain a C-type lectin domain and thus may be involved in pathogen detection. The *F41C3.8* gene encodes 2 protein isoforms between 300 and 350 amino acids in length and is predicted to be membrane localized as evidenced by a transmembrane domain.

### *mab-5* may indirectly regulate genes downstream of *S. epidermidis* exposure

Using existing ChIP-Seq datasets from the ModERN database, we cross-referenced MAB-5–binding sites with genomic coordinates of the 23 DEGs corresponding to the enriched GO terms “Stress response: C-type lectins” or “Stress response: Pathogen.” However, we did not observe any overlap between those genomic coordinates and MAB-5–binding sites. One possibility is that *mab-5* is indirectly regulating expression of these genes by working with other transcription factors to modulate the immune response following *S. epidermidis* exposure. To examine this possibility, we identified 68 transcription factors that were differentially expressed in animals infected with *S. epidermidis*, with 63 of those exclusively expressed in infected *mab-5* LOF animals. Again using existing ChIP-Seq datasets, we found a single transcription factor, PQM-1, to have binding sites overlapping 3 genes, *clec-72*, *T24B8.5*, and *F41C3.8*. Both *clec-72* and *T24B8.5* are induced during infection with multiple pathogens, whereas differential expression of *F41C3.8* after pathogen exposure has not been reported previously (Troemel *et al*. 2006; Bolz *et al*. 2010; Irazoqui *et al*. 2010; Campos *et al*. 2021). The *pqm-1* gene was upregulated in both N2 and *mab-5* GOF animals infected with *S. epidermidis* but was not differentially expressed in *mab-5* LOF animals regardless of infection status. PQM-1 has previously been implicated in the *C. elegans* innate immune response, and its function is required for the transcription of genes encoding innate immune effectors and detoxification enzymes (Tepper *et al*. 2013; Dowen *et al*. 2016; Rajan *et al*. 2019; Borbolis *et al*. 2023). Thus, our analysis of transcription factor–binding sites suggests that *mab-5* may be indirectly regulating expression of immune genes through intermediate transcription factors such as PQM-1. A limitation to our analysis is the availability of published ChIP-seq data and the experimental conditions used to obtain those data. Although we identified 68 differentially expressed transcription factors, only 18 had ChIP-Seq available for our analysis. It is entirely possible that some of the remaining 50 transcription factors could have binding sites overlapping innate immune genes similar to *pqm-1*. Additionally, the growth conditions used in the ChIP-Seq experiments could make the difference. The ChIP-Seq data used in this analysis were collected from N2 worms reared on a liquid culture diet of *E. coli* OP50 during either the embryonic or L2 larval stage of development (Niu *et al*. 2011). Our RNA-seq experiments used 1-day-old adults transferred from *E. coli* to *S. epidermidis*. It is possible that at earlier life stages and under nonpathogenic conditions, MAB-5, or any of the other transcription factors differentially expressed after *S. epidermidis* exposure, does not directly bind promoters of these immune genes but does so under different conditions.

### Integration of metabolic and innate immune pathways during microbial infection

Our findings suggest that *mab-5* is important for prolonging the lifespan of animals infected with *S. epidermidis* and may do so indirectly through intermediate transcription factors such as PQM-1. PQM-1 has prominent roles in the immune response and the metabolism of lipids in response to environmental stressors (Rajan *et al*. 2019; Heimbucher *et al*. 2020). Further, it is a key regulator of DAF-2–mediated longevity (Tepper *et al*. 2013). The involvement of PQM-1 raises the possibility that the lifespan extension following *S. epidermidis* exposure is due to a nutritional challenge rather than a robust immune response. This alternative hypothesis is supported by the differential expression of genes involved in lipid metabolism and the organismal stress response. However, it is difficult to say one way or another as microbial infections not only induce an innate immune response but also alter metabolism as the host seeks to maintain cellular homeostasis in response to the environmental stressor (Tepper *et al*. 2013). This cross-talk between innate immune and metabolic pathways is well documented in *C. elegans*, most notably in long-lived *daf-2* mutants, which require the p38 mitogen-activated protein kinase PMK-1 pathway for pathogen resistance and lifespan extension (Troemel *et al*. 2006).

To summarize, we have shown that *mab-5* is important for prolonging the lifespan of animals with an N2 genetic background following *S. epidermidis* exposure. Our transcriptomic analyses have identified genes previously implicated in immunity, such as *ech-9*, *clec-60*, *clec-71*, *dod-22*, and *F55G11.2,* as well as genes lacking functional annotation (*F18G5.6*, *F41C3.8*, *F01D5.1*, and *F28B4.3*) that are upregulated in animals expressing *mab-5* but either are not differentially expressed or are downregulated in *mab-5*(*e1239*) LOF mutants. However, it is yet to be determined how these genes are contributing to the prolonged lifespan of *mab-5*–expressing animals infected with *S. epidermidis*. Examining *S. epidermidis* susceptibility in mutant strains that express *mab-5* yet silence the expression of any of the aforementioned genes would help address this question. Further, determining the nutritional quality of *S. epidermidis* and assessing *S. epidermidis* susceptibility in insulin signaling mutants would help address whether *mab-*5 is functioning to enhance the innate immune response or is involved in the organismal response to dietary restriction. Additionally, performing ChIP-seq analysis on N2 animals and *mab-5* mutants would serve to identify downstream targets of MAB-5 following *S. epidermidis* infection. By leveraging both of these approaches, we will be able to better understand the role of the MAB-5/Hox family transcription factor in the *C. elegans* innate immune response to microbial pathogens.

## Supplementary Material

jkae054_Supplementary_Data

## Data Availability

The raw sequence files used for differential gene expression analysis are available in FASTQ format in the NCBI BioProject repository (BioProject accession number: PRJNA973087; https://www.ncbi.nlm.nih.gov/sra/PRJNA973087). Raw and processed counts of sequence reads, overlapping genes, and the test statistic and *P*-values for all genes tested can be found in a supplemental file on figshare: https://doi.org/10.25387/g3.25025117. Supplemental material available at G3 online.
