# Supplementary material for: The MAB-5/Hox family transcription factor is important for Caenorhabditis elegans innate immune response to Staphylococcus epidermidis infection
Source: G3 (Bethesda). 2024 Mar 13;14(5):jkae054. doi: 10.1093/g3journal/jkae054 (PMC11075571; doi:10.1093/g3journal/jkae054)
Supplement: jkae054_Supplementary_Data [file jkae054_supplementary_data.zip › File_S2__References_cited_in_Table_S1_-_C._elegans_and_bacterial_strains_used_G3-2024-404930.docx]

# Supplemental Material Literature Cited

1. Brenner S. The genetics of Caenorhabditis elegans. Genetics. 1974;77(1):71-94. PubMed PMID: 4366476; PubMed Central PMCID: PMCPMC1213120.

2. Kenyon C. A gene involved in the development of the posterior body region of C. elegans. Cell. 1986;46(3):477-87. doi: 10.1016/0092-8674(86)90668-9. PubMed PMID: 3731276.

3. Salser SJ, Kenyon C. Activation of a C. elegans Antennapedia homologue in migrating cells controls their direction of migration. Nature. 1992;355(6357):255-8. doi: 10.1038/355255a0. PubMed PMID: 1346230.

4. Consortium CeDM. large-scale screening for targeted knockouts in the Caenorhabditis elegans genome. G3 (Bethesda). 2012;2(11):1415-25. Epub 20121101. doi: 10.1534/g3.112.003830. PubMed PMID: 23173093; PubMed Central PMCID: PMCPMC3484672.

5. Do N, Ackley BD, Lansdon P. Draft Genome Sequence of Novel Staphylococcus epidermidis Strain EVL2000, Exhibiting Pathogenicity against Caenorhabditis elegans. Microbiol Resour Announc. 2022;11(4):e0123921. Epub 20220314. doi: 10.1128/mra.01239-21. PubMed PMID: 35286161; PubMed Central PMCID: PMCPMC9022524.
