# Supplementary material for: The MAB-5/Hox family transcription factor is important for Caenorhabditis elegans innate immune response to Staphylococcus epidermidis infection
Source: G3 (Bethesda). 2024 Mar 13;14(5):jkae054. doi: 10.1093/g3journal/jkae054 (PMC11075571; doi:10.1093/g3journal/jkae054)
Supplement: jkae054_Supplementary_Data [file jkae054_supplementary_data.zip › Table_S1__Strain_name_and_genotype_for_the_C._elegans_and_bacterial_G3-2024-404930.docx]

| **Supplemental Table 1: *C. elegans* and bacterial strains used in this study** | | |
| --- | --- | --- |
| Strain | Relevant Properties | Reference or Source |
| *Caenorhabditis elegans* |  |  |
| N2 | var. Bristol wild-type | [1] |
| LE2961 | *mab-5*(*e1239*) | [2] |
| LE2467 | *mab-5*(*gk670*) | [3] |
| CB3256 | *mab-5*(*e1751*) | [4] |
|  |  |  |
| Bacterial Strains |  |  |
| *E. coli* OP50 | uracil-requiring mutant | [1] |
| *S. epidermidis* EVL2000 | Isolated as a laboratory contaminant | [5] |
