# Supplementary material for: The MAB-5/Hox family transcription factor is important for Caenorhabditis elegans innate immune response to Staphylococcus epidermidis infection
Source: G3 (Bethesda). 2024 Mar 13;14(5):jkae054. doi: 10.1093/g3journal/jkae054 (PMC11075571; doi:10.1093/g3journal/jkae054)
Supplement: jkae054_Supplementary_Data [file jkae054_supplementary_data.zip › Table_S2__All_trials_of_survival_data_for_N2_and_mab-5_mutants_on_E._coli_OP50_G3-2024-404930.docx]

| **Supplemental Table 2: Median survival of pathogen susceptibility experiments** | | | |
| --- | --- | --- | --- |
| Experiment | Trial | Total worms | Median survival (days) |
| *E.coli* OP50 |  |  |  |
| N2 | 1 | 30 | 8 |
| N2 | 2 | 30 | 11 |
| N2 | 3 | 30 | 11 |
|  |  |  |  |
| LE2961 *mab-5* (*e1239*) | 1 | 30 | 12 |
| LE2961 *mab-5* (*e1239*) | 2 | 30 | 9 |
| LE2961 *mab-*5(*e1239*) | 3 | 30 | 12 |
|  |  |  |  |
| LE2467 *mab-5* (*gk670*) | 1 | 30 | 9 |
| LE2467 *mab-5* (*gk670*) | 2 | 30 | 6 |
| LE2467 *mab-5* (*gk670*) | 3 | 30 | 11 |
|  |  |  |  |
| CB3256 *mab-5*(*e1751*) | 1 | 30 | 8 |
| CB3256 *mab-5*(*e1751*) | 2 | 30 | 8 |
| CB3256 *mab-5*(*e1751*) | 3 | 30 | 10 |
|  |  |  |  |
| *S. epidermidis* (EVL2000) |  |  |  |
| N2 | 1 | 30 | 14 |
| N2 | 2 | 30 | 14 |
| N2 | 3 | 30 | 17 |
| N2 | 4 | 30 | 19 |
|  |  |  |  |
| LE2961 *mab-5* (*e1239*) | 1 | 30 | 12 |
| LE2961 *mab-5* (*e1239*) | 2 | 30 | 5 |
| LE2961 *mab-*5(*e1239*) | 3 | 30 | 14 |
| LE2961 *mab-5*(*e1239*) | 4 | 30 | 11 |
|  |  |  |  |
| LE2467 *mab-5*(*gk670*) | 1 | 30 | 13 |
| LE2467 *mab-5*(*gk670*) | 2 | 30 | 7 |
| LE2467 *mab-5*(*gk670*) | 3 | 30 | 12 |
| LE2467 *mab-5*(*gk670*) | 4 | 30 | 14 |
|  |  |  |  |
| CB3256 *mab-5*(*e1751*) | 1 | 30 | 14 |
| CB3256 *mab-5*(*e1751*) | 2 | 30 | 15 |
| CB3256 *mab-5*(*e1751*) | 3 | 30 | 16 |
|  |  |  |  |
